# Supplementary material for: Drug specificity and affinity are encoded in the probability of cryptic pocket opening in myosin motor domains
Source: eLife. 2023 Jan 27;12:e83602. doi: 10.7554/eLife.83602 (PMC9995120; doi:10.7554/eLife.83602)
Supplement: Supplementary file 1. [file elife-83602-supp1.docx]

**Supporting Information for**

Drug specificity and affinity are encoded in the probability of cryptic pocket opening in myosin motor domains

Artur Meller, Jeffrey M. Lotthammer, Louis G. Smith, Borna Novak, Lindsey A. Lee, Catherine C. Kuhn, Lina Greenberg, Leslie A. Leinwand, Michael J. Greenberg, Gregory R. Bowman

This PDF includes:

Supplementary Files 1A to 1C

**Supplementary File 1A: IC50 values for different myosin isoforms**

| **Isoform** | **Species** | **IC50/Ki [μM]** | **Citation** |
| --- | --- | --- | --- |
| Skeletal Muscle Myosin II | Rabbit | 0.11 | Varkuti et. al. |
| Fast skeletal | Rabbit | 0.5 | Limouze et. al. |
| Skeletal Muscle Myosin II | Rabbit | 0.28 | Radnai et. al. |
| β-cardiac | Porcine | 1.2 | Limouze et. al. |
| β-cardiac | Porcine | 1.9 | Radnai et. al. |
| Nonmuscle Myosin IIA | Human | 5.1 | Limouze et. al. |
| Nonmuscle Myosin IIA | Human | 3.58 | Zhang et. al. |
| Nonmuscle Myosin IIA | Human | 2.9 | Radnai et. al. |
| Unphosphorylated Smooth Muscle Myosin II | Chicken | 17.5 | Wang et. al. |
| Unphosphorylated Smooth Muscle Myosin II | Bovine | 10.1 | Wang et. al. |
| Phosphorylated Smooth Muscle Myosin II | Chicken | 23.5 | Wang et. al. |
| Smooth Muscle Myosin | Chicken | 6.47 | Zhang et. al. |
| Smooth Muscle Myosin 2A and 2B | Chicken | 3 | Eddinger et. al. |
| Smooth Muscle Myosin | Turkey | 79.6 | Limouze et. al. |
| Smooth Muscle Myosin II | Chicken | 3.2 | Radnai et. al. |

**Supplementary File 1B: Percent identity in motor domain sequence between myosin-II isoforms in this study**

|  | MYH11 | MYH9 | MYH7b | MYH7 | MYH2 |
| --- | --- | --- | --- | --- | --- |
| MYH11 | 100 | 84.58 | 52.03 | 52.45 | 51.56 |
| MYH9 | 84.58 | 100 | 51.01 | 52.23 | 50.67 |
| MYH7b | 52.03 | 51.01 | 100 | 69.65 | 66.24 |
| MYH7 | 52.45 | 52.23 | 69.65 | 100 | 80.77 |
| MYH2 | 51.56 | 50.67 | 66.24 | 80.77 | 100 |

**Supplementary File 1C: Structural similarity between myosin-II prepowerstroke state crystal structures as assessed by C-α root mean square deviation**

Units of Å

|  | 5N6A | 1BR2 | 5I4E |
| --- | --- | --- | --- |
| 5N6A | - | 0.75 | 0.79 |
| 1BR2 | 0.75 | - | 0.57 |
| 5I4E | 0.79 | 0.57 | - |
